# Visualization of IOL Material-Induced Changes in Retinal Color Stimulus

**DOI:** 10.1155/2016/4680621

**Published:** 2016-06-28

**Authors:** Stephan Reiss, Karsten Sperlich, Martin Kunert, Rudolf F. Guthoff, Heinrich Stolz, Anselm Jünemann, Oliver Stachs

**Affiliations:** ^1^Institute of Physics, University of Rostock, Albert-Einstein-Straße 23-24, 18059 Rostock, Germany; ^2^Department of Ophthalmology, University of Rostock, Doberaner Straße 140, 18057 Rostock, Germany; ^3^Institute for Biomedical Engineering, University of Rostock, Friedrich-Barnewitz-Straße 4, 18119 Rostock, Germany

## Abstract

*Purpose*. Different IOL materials, particularly blue-light filtering materials, have different spectral transmittance characteristics. The color stimuli, which influence retinal receptors objectively, have consequently implications for color perception. We report on the quantitative determination of IOL-specific transmittance characteristics and present a method visualizing the resultant changes in color stimulus.* Methods*. A setup was realized to quantify IOL-absorption in a range of 390–780 nm. To visualize the influence of the different spectral transmittance characteristics an algorithm was developed, which converts RGB-pixel values of images into spectra, which performs the corresponding transmittance correction, reconverts to RGB, and reconstructs the image. IOLs of hydrophobic acrylate and hydrophilic acrylate with a hydrophobic surface in each case with/without blue-light filter were examined.* Results*. Assessment of the reference images verifies the suitability of the pipeline. Evaluation of the transmittance spectra reveals differences of material- and manufacturer-specifics, which are capable of inducing considerable changes in color perception, particularly in the blue color range and mixed colors involving blue.* Conclusions*. The developed technique provides an approach for determining IOL-specific transmittance behavior and subsequently its influence on the retinal color stimulus. Problems of altered color perception are occasionally reported after cataract surgery and these become obvious with the visualization procedure developed here.

## 1. Introduction

Color perception is a complex process in which a chain of signals consisting of physical, physiological, and psychological components evokes the actual impression of color. Radiation in a wavelength range from 390 nm to 780 nm is designated as visible light. The relative spectral distribution of the primary light sources or light bodies in the perception of color will be affected by the respective spectral transmittance characteristics of the individual optically refracting media of the eye, namely, the cornea, aqueous humor, lens, and vitreous humor. A color stimulus is induced on the retina and is converted by the specific response of the retina into a color intensity.

Cataract surgery using intraocular lenses (IOLs) with different spectral transmittance characteristics is known to affect the retinal color stimulus. This effect is to be expected in the light of previously published IOL transmittance curves [1–3], and yet its impact is difficult to quantify. The present study therefore sets out to calculate the changes in color stimuli on the retina by quantitatively determining the spectral transmittance characteristics of IOLs made from different materials and supplied by different manufacturers. The main aim of this study was to understand how IOL implantation using different optic materials influences the retinal color stimulus.

## 2. Method

To visualize the retinal color stimulus change due to the spectral transmittance characteristics of different IOL materials, it is necessary to determine transmittance in the wavelength range of visible light.

### 2.1. Transmittance Measurement

**Figure 1:**
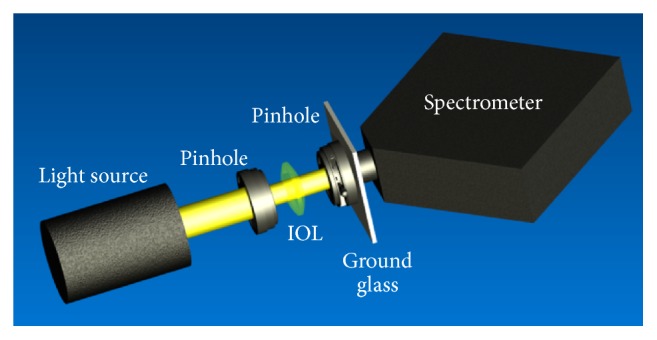
Setup for determining transmittance properties.

With a light source (KL1500, Schott, Mainz/Germany) radiating in a wavelength range from 390 nm to 780 nm, two pinhole apertures ensured that transmittance was measured through the IOL in a diameter of 4.5 mm (Figure 1). A ground glass (SM5, Edmund Optics, Karlsruhe/Germany) was used to generate homogeneous light distribution and to bypass any influence of IOL focus. A high-resolution spectrometer (HR4000, Ocean Optics, Dunedin, FL/USA) was used to determine spectral intensity distribution in the investigated wavelength range.

The spectral transmittance curve *T*(*λ*) is a relative quantity. In an initial step the spectral intensity of the light source was measured, background-corrected, and normalized to *I*_*L*_(*λ*) as reference.

In the next step the intensity spectrum with IOL inserted was measured, background-corrected, and scaled using the same normalization factor to obtain *I*_IOL_(*λ*). The spectral transmittance curve *T*(*λ*) was then defined as the ratio of *I*_IOL_(*λ*) divided by *I*_*L*_(*λ*).

However, this approach was invalidated because the focusing characteristic of the IOL resulted in an incorrect intensity spectrum of transmitted light compared with the spectrum of the light source. This potentially leads to a transmittance greater than 1, which is contrary to the laws of physics.

**Figure 2:**
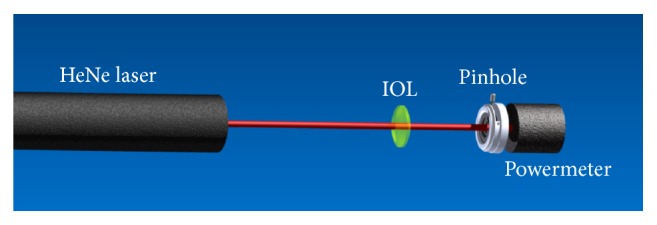
Schematic illustration of the experimental setup for normalization of the intensity spectrum with IOL (for the wavelength 633 nm). The pinhole ensured that no stray light was incident on the power meter (Powermeter 1918-R, Newport Corporation, Irvine, CA/USA).

To circumvent this problem we needed to scale *I*_IOL_(*λ*) correctly. With the aid of a HeNe laser at 633 nm and a powermeter, we determined transmittance *T*_PM_ at this specific wavelength. Since the powermeter has a large detector area, this measurement has the advantage of not being affected by the focusing characteristic of the lens, compared with the entrance slit of a spectrometer, and hence permits laser light to be collected entirely (see Figure 2).

For *λ* = 633 nm the scaling parameter *S* can easily be calculated from *T*_PM_ = *SI*_IOL_/*I*_*L*_. The following general equation is therefore valid:

(1) $$\begin{array}{c} T\left(\;\lambda \;\right)=\frac{SI_{\text{IOL}}\left(\lambda \right)}{I_{L}\left(\lambda \right)}. \end{array}$$

**Figure 3:**
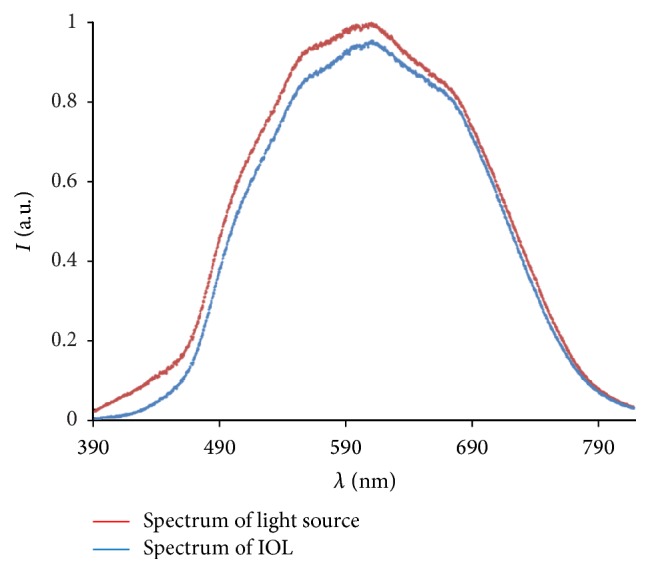
Background-corrected and normalized intensity distributions in the wavelength range from 390 nm to 780 nm for the light source (red) only and an IOL example (Hoya NY60) (blue).

**Figure 4:**
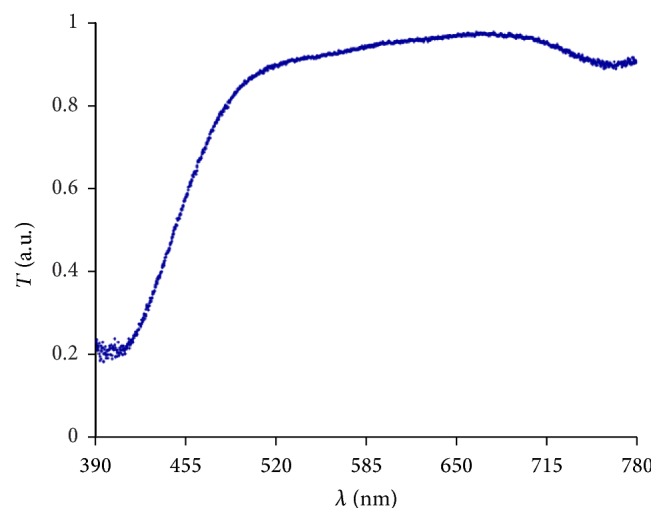
Example of a measured transmittance curve for an IOL (Hoya NY60).

Figure 3 illustrates the spectrum of the light source *I*_*L*_(*λ*) and the now correctly scaled spectrum of light transmitted through the IOL *I*_IOL_(*λ*), while the derived transmittance *T*(*λ*) is depicted in Figure 4.

### 2.2. Visualization

We set out to visualize the retinal color stimulus changes induced by the measured transmittance spectra of the artificial IOLs. The analysis involved calculating the influence of the IOL-specific transmittance on a reference image on a monitor. Each pixel corresponds to a triple of color values, namely, Red Green Blue (RGB), and has to be recalculated according to the measured transmittance curve. To apply the RGB value of a pixel to the transmittance spectrum, this must be transformed into the corresponding wavelength. The literature contains equations that enable wavelength values to be converted to RGB values [4]. However, these equations are not suitable since each pixel corresponds to a spectrum instead of one single wavelength, a disadvantage that is especially obvious for mixed colors, such as pink or white. In order to obtain a spectrum for each pixel we adopted a different approach.

**Figure 5:**
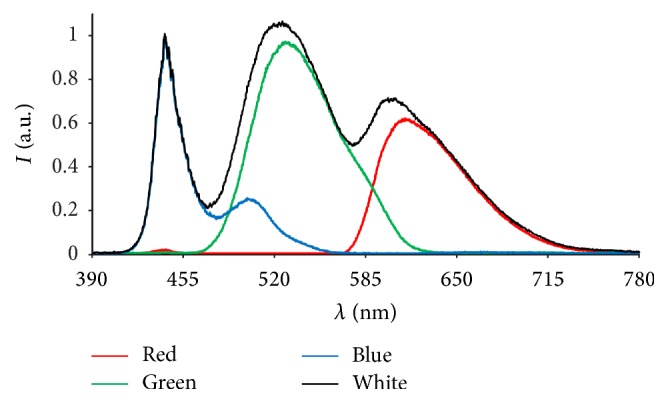
Intensity spectra of RGB colors and white of an LED monitor (Flatron IPS235P, LG Electronics Inc., Seoul, South Korea) measured with a spectrometer (HR4000, Ocean Optics, Dunedin, FL/USA).

A spectrometer (HR4000, Ocean Optics, Dunedin, FL/USA) was used to measure separately the intensity spectrum of each RGB color of a monitor (Flatron IPS235P, LG Electronics Inc., Seoul, South Korea) (Figure 5). The most intense color (blue) was selected to be normalized to 1 and the other two colors were normalized using the same factor. Addition of these three spectra yielded the spectrum for white. Measuring the spectrum for white itself yielded the same spectrum and, since the two curves were identical, we plotted only one curve for white.

Having identified a spectrum for each color, a spectrum for every RGB value was obtained simply by multiplying the spectra for red, green, and blue with the corresponding RGB value and summing these spectra. This yielded a spectrum for each pixel of the reference image and each pixel spectrum could then be multiplied by the spectral transmittance of the specific IOL. Under these conditions the pixel spectra are altered in the same way as would be experienced by a patient with an implanted IOL. The final task was to identify the new RGB values corresponding to the new modified pixel spectra. Since the spectra for red, green, and blue are of fixed shape, it is impossible to achieve RBG values that match the new spectrum exactly. Therefore we implemented an iterative algorithm in Mathematica 10.0 (Wolfram Research, Champaign, IL/USA) to identify the RGB set whose pixel spectrum best matched the modified pixel spectrum. These values correspond to the transmittance properties of the specific IOL. This approach allowed visualization of the change in retinal receptor color stimulus caused by an implanted artificial lens.

### 2.3. Setup Verification

In a subsequent verification step the conversion algorithm and the experimental setup were tested using a neutral density filter. With its known spectral transmittance of 70% it is obvious how the “modified” image has to look like. This is a simple way to test whether or not the program and our experimental setup were operating as intended. Subsequently, the same routine was applied to the reference image using IOL transmittance curves.

### 2.4. Material Selection

The color stimulus change induced by IOLs from different materials and manufacturers (Alcon, Hoya, Zeiss, Polytech, Domilens, and Medicontur) was investigated according to the procedure outlined above. IOL selection was performed to address the influence of hydrophobic acrylate materials and hydrophilic acrylate materials with a hydrophobic coating. The following material groups were investigated:

1. Hydrophobic acrylate materials without blue-light filter (Hoya VA-65BB, Alcon MA60AC).
2. Hydrophobic acrylate materials with blue-light filter (Alcon SN60AT, Hoya NY60).
3. Hydrophilic acrylate with a hydrophobic coating without blue-light filter (Zeiss 209M, Polytech Opti Vis).
4. Hydrophilic acrylate with a hydrophobic coating with blue-light filter (Domilens Domicryl 677ABY, Medicontur 677MY).

## 3. Results

**Figure 6:**
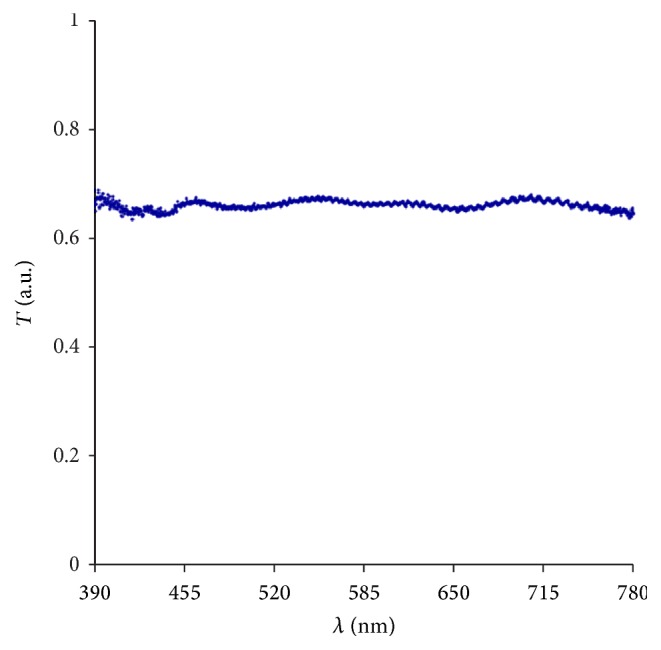
Measured transmittance spectrum of the 70% neutral density filter.

Figure 6 shows the transmittance curve of the neutral density filter. As anticipated, an almost constant transmittance pattern was recorded across the entire wavelength range from 390 nm to 780 nm.

**Figure 7:**
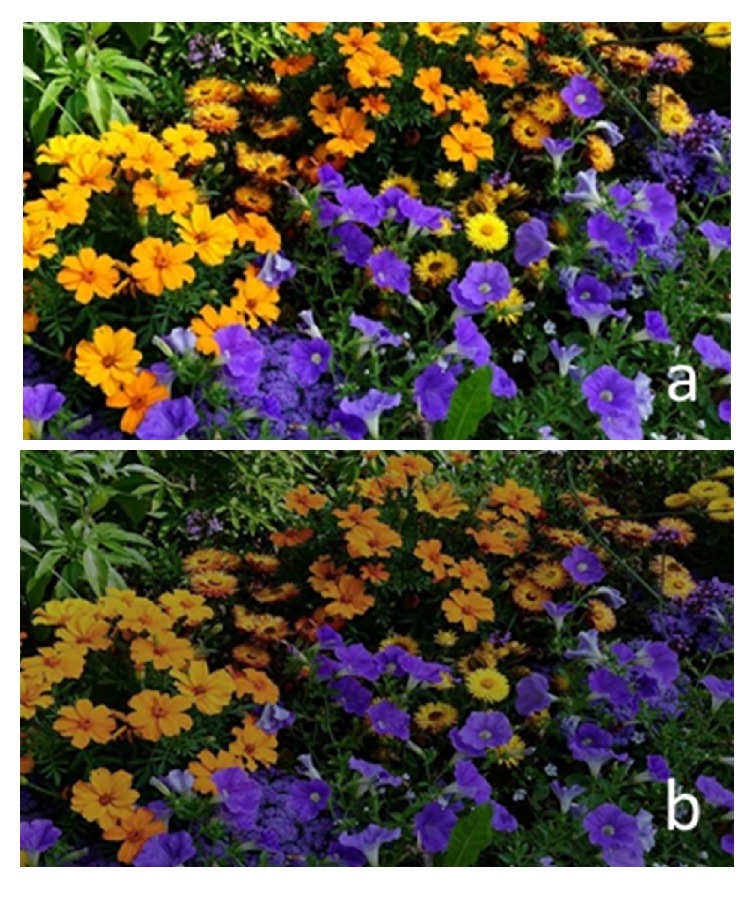
Effect of neutral density filter transmittance on the reference image: (a) reference image and (b) reference image affected by neutral density filter transmittance curve.

As expected, there was no selective change in brightness of any one color or wavelength. Instead, the brightness of the entire image was reduced (by about 30%) (Figure 7).

**Figure 8:**
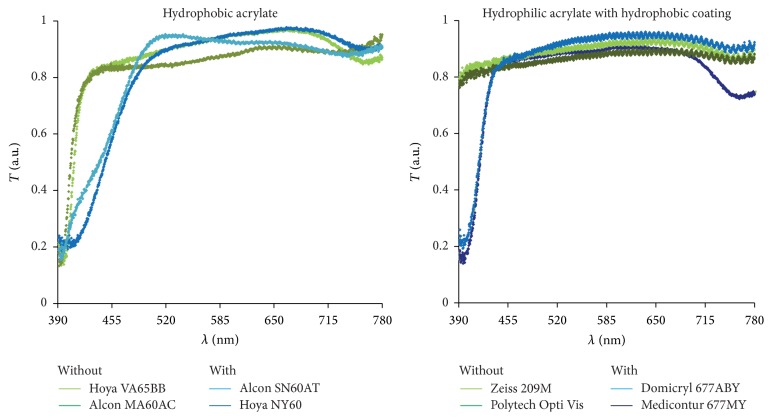
Transmittance spectra of IOLs made from different materials with (blue) and without blue-light filter (green).

On the basis of these validating results, the IOL transmittance spectra were measured (see Figure 8).

The two raw materials tested (hydrophobic acrylate, hydrophilic acrylate with a hydrophobic coating) are both manufactured with and without blue-light filter. All lenses showed similar transmittance curves with more or less constant transmittance between 480 nm and 700 nm and a decline of 60% below 480 nm, except IOLs made from hydrophilic acrylate with a hydrophobic coating without blue-light filter.

In the wavelength range from 390 nm to 780 nm the hydrophilic IOLs with a hydrophobic coating without blue-light filter showed an almost constant high transmittance of about 80% to 90%. The hydrophobic lenses without blue-light filter had a transmittance edge at about 410 nm; at that point transmittance increased from about 20% within 20 nm to 80% and then remained relatively constant at about 95%.

The hydrophobic IOLs with blue-light filter revealed a transmittance of 20% at 390 nm. Over a wavelength range of roughly 110 nm the intensity of the transmitted radiation through the lens increased to approximately 95%. However, the hydrophilic IOLs with a hydrophobic coating and blue-light filter achieved this change in transmittance within a wavelength range of approximately 40 nm.

**Figure 9:**
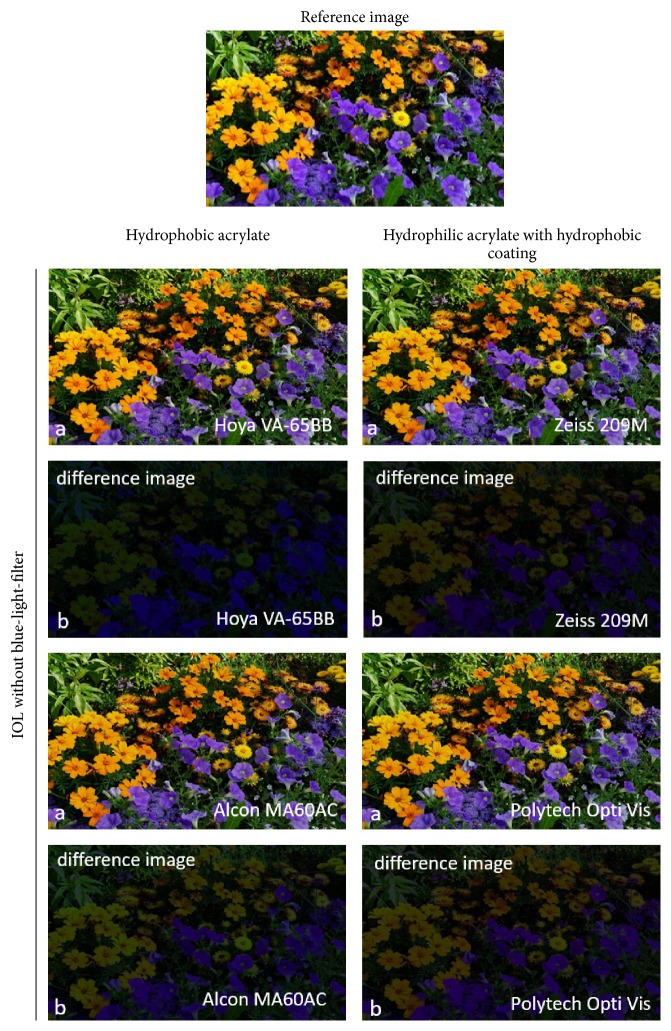
(a) Influence of the specific transmittance curves on the reference image for hydrophobic IOL materials and hydrophilic IOL materials with a hydrophobic coating, in each case without blue-light filter. (b) Difference between reference image and the image affected by a specific transmittance curve for each IOL (for enhanced visualization, the brightness value was increased by a factor two).

**Figure 10:**
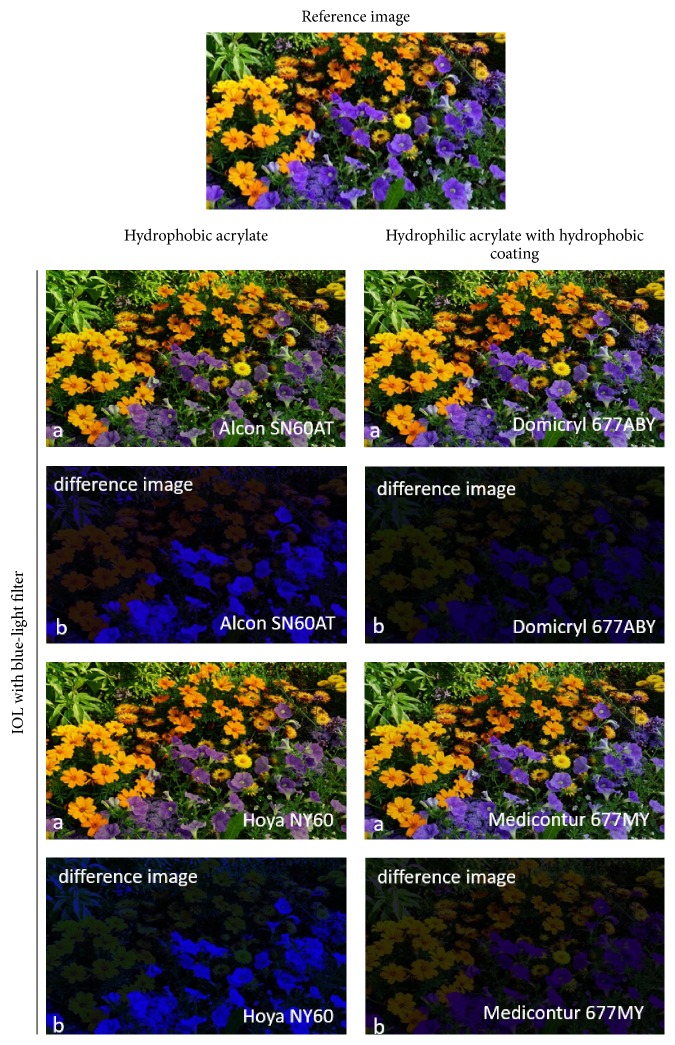
(a) Influence of the specific transmittance curves on the reference image for hydrophobic IOL materials and hydrophilic IOL materials with a hydrophobic coating, in each case with blue-light filter. (b) Difference between reference image and the image affected by a special transmittance curve for each IOL (for enhanced visualization, the brightness value was increased by a factor two).

Visualization of retinal color stimuli changes, based on the IOL-specific transmittance curves, was determined and is shown in Figures 9 and 10. The transmittance characteristics were applied to the reference image according to the conversion algorithm described above. Minimal color stimulus changes were found to be induced by hydrophobic acrylate without blue-light filter as well as by hydrophilic acrylate with a hydrophobic coating without and with blue-light filter (Figures 9 and 10, resp.). Hydrophobic acrylate lenses with blue-light filter, however, showed a significant change, especially in the blue color range (Figure 10).

These results were also evident in the representation of the difference images. A black difference image signifies that there was no change at all. However, intense blue regions, for example, denote a major change in the blue wavelength range due to the IOL. The more intense a color is, the more it is affected by the IOL. The intensity of difference images has been increased by a factor of two for enhanced visualization of the differences.

## 4. Discussion

The aim of the present study was to visualize changes in retinal color stimulus induced by different lens materials, including the effect of incorporated blue-light filters. For this purpose, a simple measurement method was developed to determine the transmittance behavior of IOLs in the wavelength range from 390 nm to 780 nm. Since spectral transmittance was determined as a percentage value (1 corresponds to 100%), specific IOL design parameters such as thickness or refractive index are no longer important in our measurement. Initial experiments with a neutral density filter demonstrated the validity of the experimental setup and the visualization algorithm.

As illustrated in Figure 8, different material- and manufacturer-dependent transmittance characteristics were detected. IOLs manufactured from the same material (hydrophobic acrylate or hydrophilic acrylate with a hydrophobic coating) by the same manufacturer exhibited identical transmittance behavior (data not presented) whereas divergent patterns may be present between different manufacturers. For example, Alcon SN60AT and Hoya NY60 are both manufactured from hydrophobic acrylate and include a blue-light filter, but their transmittance behaviors differed. A similar difference was found between Domilens Domicryl 677ABY and Medicontur 677MY for hydrophilic acrylate with a hydrophobic coating. Surprisingly, these different transmittance behaviors have a negligible effect on the retinal color stimulus, as shown in Figure 10.

In general, in the range from 390 nm to 500 nm, hydrophilic acrylate IOLs with a hydrophobic coating (with or without blue-light filter) are characterized by significantly better transmittance than hydrophobic acrylate lenses. They display almost constantly high transmittance of about 85% across the entire wavelength range of the visible spectrum. This property can also be recognized in the visualization of the color stimulus.

The blue-light filter influence on the retinal color stimulus is not insignificant. The transmittance curves of the IOLs tested show the expected difference between materials with and without blue-light filter. Both material groups (hydrophobic acrylate, hydrophilic acrylate with a hydrophobic coating) show lower transmittance in the range from 390 nm to 450 nm when the IOLs have a blue-light filter: similar findings have already been published elsewhere [1–3].

This difference has an effect in terms of the retinal color stimulus. In the case of hydrophilic lenses with a hydrophobic coating with and without blue-light filter, the visualization of transmittance curve effects on the reference image (see Figures 9 and 10) shows that there is only a small change in color stimulus compared with the initial reference image. This is confirmed in the difference images where all colors are represented almost equally dark or equally bright, respectively.

In contrast, transmittance differences between hydrophobic acrylate IOLs with blue-light filter (Alcon SN60AT, Hoya NY60) and without blue-light filter (Hoya VA-65BB, Alcon MA60AC) are responsible for significant color stimulus changes (see Figures 9 and 10). In particular, hydrophobic acrylate IOLs with blue-light filter provide a significant change in blue and mixed colors involving blue compared with hydrophobic acrylate IOLs without blue-light filter.

The calculated difference images reflect this. Both investigated hydrophobic acrylate IOLs without blue-light filter reveal approximately the same brightness changes in the difference images. However, the difference images for hydrophobic IOLs with blue-light filter show an increased brightness level for blue, which indicates a strong influence on the blue-affected colors due to the transmittance characteristics of these IOLs. The blue color stimulus changes considerably, while the other colors of the visible spectrum remain unaffected. In this context, it is understandable that other studies have demonstrated generally larger color discrimination with blue-light filter IOLs than with clear IOLs [5–10].

The present study also has some limitations. The influence of the cornea, aqueous humor, and vitreous body has not been investigated. However, their transmittances do not change on IOL implantation and they can be considered as constant factors having no influence on the color stimulus.

It is worth mentioning that our method implies the use of continuous spectra without spectral intensity peaks or jumps; that is, a patient looking at an image on a present-day LED monitor fulfills the continuous spectrum requirement.

Furthermore, the stimulus on the retina does not equate to color perception processed by the visual pathway. We might also be criticized for not investigating IOL transmittance behavior in the UV-A (315 nm–380 nm) and UV-B (280 nm–315 nm) ranges. The cornea, aqueous fluid, and vitreous body absorb most ultraviolet radiation below 300 nm in the phakic eye. Moreover, information between 300 nm and 380 nm does not contribute to color vision and our investigation relates only to IOL material-induced changes in retinal color stimulus. Protection or safety aspects are not addressed in this study because the transmittance behavior of IOLs in the UV-A and UV-B ranges has already been investigated by Laube et al. [2].

## 5. Summary

To the best of our knowledge this is the first study to investigate the implications of different IOL materials on color perception. The technique developed here provides a simple approach for determining IOL-specific transmittance behavior and subsequently its influence on the retinal color stimulus. Problems of altered color perception are occasionally reported by patients after cataract surgery and these become obvious and understandable with the visualization procedure developed here. Major differences exist between IOL optic materials and these may be responsible for not insignificant changes in retinal color stimulus. For blue-light filter IOLs, due to their relatively constant transmittance of about 85% in the visible wavelength range, hydrophilic acrylate IOLs with a hydrophobic coating have significantly less influence on retinal color stimulus than hydrophobic acrylate IOLs. In summary, IOLs of any manufacturer without blue-light filter have no significant influence on the retinal color stimulus.
